# Perspectives on Health Logistics in Saudi Arabia: Progress and Challenges

**DOI:** 10.3390/healthcare14213831

**Published:** 2026-11-09

**Authors:** Haytham A. Sheerah, Meraee Aljabri, Shouq Alzaaqi, Emad Almohammadi, Hebah Dada, Abdulrhman I. Mohana, Asmaa M. Altamimi, Khaled Alamri, Shahah Abu Thnain, Ashraf A. AlGrain, Ahmed Al-Jedai, Duncan Selbie

**Affiliations:** 1 International Collaboration Department, Chief Executive Office, Public Health Authority, Riyadh 13351, Saudi Arabia; 2 Population Health Deputyship, Ministry of Health, Riyadh 11176, Saudi Arabia; 3 Preventive Medicine and Public Health Department, Faculty of Medicine, King Abdul-Aziz University, Jeddah 22254, Saudi Arabia; 4 Deputyship of Nationalization & Human Capital, Ministry of Health, Riyadh 11176, Saudi Arabia; 5 King Abdullah International Medical Research Center, King Saud bin Abdulaziz University for Health Sciences, Ministry of National Guard Health Affairs, Riyadh 11426, Saudi Arabia; 6 Communicable Diseases Control Sector, Public Health Authority, Riyadh 13351, Saudi Arabia; 7 General Department of Policies, Saudi Commission for Health Specialities, Riyadh 12511, Saudi Arabia; 8 Public Health Laboratories Sector, Public Health Authority, Riyadh 13351, Saudi Arabia; 9 Deputyship of Supply Chain, Ministry of Health, Riyadh 11176, Saudi Arabia; 10 Development of Health Care Sectors at Local Content & Government Procurements Authority (LCGPA), Riyadh 12752, Saudi Arabia; 11 Colleges of Medicine and Pharmacy, Al-Faisal University, Riyadh 11533, Saudi Arabia; 12 Saudi Society of Clinical Pharmacy, Riyadh 11614, Saudi Arabia; 13 Public Health Authority, Riyadh 13351, Saudi Arabia; 14 International Association of National Public Health Institutes, Robert Koch Institute, 13353 Berlin, Germany

**Keywords:** health supply chain, supply chain resilience, centralized procurement, local pharmaceutical manufacturing, Vision 2030, Saudi Arabia

## Abstract

Saudi Arabia has identified healthcare supply chain transformation as a key component of the Health Sector Transformation Program under Vision 2030, yet evidence on these reforms remains fragmented across sectors and policy domains. Drawing on the published literature, policy documents, institutional reports, and the authors’ experience in national procurement, regulation, and public health, this perspective examines four interacting components of transformation—procurement governance, digital infrastructure, local manufacturing capacity, and emergency preparedness—and applies this framework to antimicrobial resistance. Substantial reforms have been implemented, notably centralized procurement through the National Unified Procurement Company, national digital surveillance and traceability systems, and the localization of strategic medicines such as insulin. We propose that interactions among these reforms may influence supply chain resilience, although weak public–private data integration, dependence on imported active pharmaceutical ingredients, and limited performance evaluations prevent firm conclusions about their system-level effects. Remaining challenges include recurrent medicine shortages, regulatory bottlenecks, workforce gaps, and the limited availability of public performance indicators. The Saudi experience suggests that centralized, state-led reform should balance efficiency with redundancy, prioritize localization selectively, and build transparent performance monitoring into reforms from the outset.

## 1. Introduction

Robust and resilient health supply chains are essential components of healthcare systems because they ensure the availability of medicines, vaccines, diagnostics, and medical supplies needed for routine care and public health emergencies [1]. Recent global disruptions, particularly the COVID-19 pandemic, have highlighted the vulnerability of healthcare supply chains and reinforced the importance of strengthening procurement systems, logistics infrastructure, supply chain governance, and emergency preparedness [2,3,4]. In response, the World Health Assembly has called on Member States to strengthen local production of medicines and other health technologies to improve access [5].

In Saudi Arabia, healthcare supply chain reform has become an important component of the Health Sector Transformation Program (HSTP) under Vision 2030 [6]. Recent initiatives have focused on centralized procurement, digital supply chain platforms, expansion of cold chain infrastructure, and the localization of pharmaceutical and medical device manufacturing [7,8,9,10]. However, several challenges remain, including recurrent medicine shortages linked to procurement and regulatory weaknesses, heavy reliance on imports, and broader supply chain risks [11,12,13].

The existing literature has primarily focused on individual components of the Saudi healthcare supply chain, with limited attention to the interrelationships between procurement, digital transformation, emergency preparedness, local manufacturing, and health security policies. Therefore, this review provides an integrated perspective on health supply chain transformation in Saudi Arabia, examining not only individual reforms but also how they interact, reinforce, or constrain one another, and identifying the key challenges that continue to limit system performance and resilience.

## 2. Approach and Sources

This article is a perspective-based review that draws on the authors’ experience in national health procurement, pharmaceutical regulation, public health, and local content policy, complemented by a non-systematic review of published literature and official documents. The primary purpose is to provide a policy-oriented interpretation of healthcare supply chain reforms, informed by published evidence and professional experience, rather than to produce a comprehensive synthesis of the literature or a quantitative evaluation of reform outcomes.

Peer-reviewed studies were identified through searches of PubMed and Scopus conducted during October 2025, using combinations of terms such as “Saudi Arabia,” “health supply chain,” “healthcare procurement,” “cold chain,” “local pharmaceutical manufacturing,” “medicine shortages,” and “antimicrobial resistance,” supplemented by screening the reference lists of relevant publications (Appendix A).

We focused on publications from January 2016, when Vision 2030 was launched, to October 2025; earlier sources were used only for historical background. Searches were limited to publications in English, and official documents were consulted in English or Arabic. We obtained policy documents, regulatory frameworks, and institutional reports from the Ministry of Health, the National Unified Procurement Company (NUPCO), the Saudi Food and Drug Authority (SFDA), the Local Content and Government Procurement Authority (LCGPA), and the Public Health Authority. A small number of industry and market reports were used only to describe market context.

Sources were selected based on their relevance to Saudi healthcare supply chain reforms or challenges in procurement, digital systems, emergency preparedness, local manufacturing, or antimicrobial resistance (AMR). Broader international sources were included where necessary to provide policy or conceptual background. Within these topics, priority was given to sources documenting implemented initiatives, supply chain challenges, or measured outcomes. Sources fell into four categories: (i) peer-reviewed studies; (ii) official government, institutional, or international organization documents; (iii) news reports of official announcements; and (iv) commercial market reports. Two authors (H.A.S. and M.A.) assessed relevance using these criteria. Co-authors with expertise in procurement, regulation, public health, and local content reviewed the sections relevant to their fields, verified institutional information, and suggested additional official sources.

Information was organized deductively into the four components, which were derived from the reform pillars of the Health Sector Transformation Program and the mandates of the bodies responsible for them. For each component, we distinguished reforms that have been implemented, their expected benefits, and outcomes that have been measured. In judging reliability, we gave priority to peer-reviewed studies and official documents. News reports were used only to document announced events such as facility openings or agreements, and commercial market reports only to describe market context. Neither was used to support conclusions about supply chain performance or resilience, and both are identified as such in the text. No formal screening protocol, quality scoring, or quality appraisal was applied, and the review is therefore not intended to be exhaustive.

## 3. Conceptual Framework

Health supply chain transformation in Saudi Arabia can be understood through four interacting components: procurement governance, digital infrastructure, local manufacturing capacity, and emergency preparedness mechanisms. These components align with the Health Sector Transformation Program’s main reform pillars and the mandates of key national bodies, including NUPCO, SFDA, LCGPA, and the Public Health Authority [6,7,14]. They are not independent. Procurement governance shapes incentives for domestic manufacturing by determining how demand is organized; digital infrastructure provides the information flows that connect procurement, distribution, and emergency response; local manufacturing determines upstream supply security; and emergency preparedness determines the capacity to absorb sudden shocks. Drawing on health system resilience frameworks [15,16], we view resilience as a property that emerges from the linkages among these components rather than from any single reform.

Central to this framework is the distinction between procurement efficiency and supply chain resilience. Centralized procurement may improve purchasing efficiency through demand aggregation and standardized tendering, but these benefits do not necessarily translate into greater supply continuity during disruptions. In particular, reliance on a limited number of suppliers or centralized purchasing pathways may increase vulnerability when alternative sources are unavailable. Conversely, maintaining multiple suppliers, emergency purchasing arrangements, and strategic reserves may improve resilience but involve additional costs and operational complexity [15,16].

We synthesized the evidence by comparing documented policy initiatives with findings from empirical studies and identifying areas where performance outcomes had not been evaluated. Relationships between the four reform components (Procurement governance, digital infrastructure, emergency preparedness, and local manufacturing capacity) were then interpreted using the existing health system resilience literature. The authors’ professional experience informed the interpretation of institutional arrangements and practical challenges but was not treated as independent evidence of reform effectiveness.

## 4. Elements of Health Supply Chain Transformation

For each component, we summarize the reforms implemented, their expected benefits, and the outcomes documented to date (Table 1).

### 4.1. Centralized Procurement and Standardization

Centralized procurement has become a major component of healthcare reform in Saudi Arabia under Vision 2030. A central element of this strategy is NUPCO, which was established to coordinate the procurement and supply of pharmaceuticals, medical devices, and medical consumables across the public healthcare sector [7]. This approach replaced the previously fragmented procurement model in which purchasing decisions were largely undertaken at the institutional or regional level [17].

The centralized procurement model is designed to consolidate demand, support bulk purchasing, and improve coordination across healthcare institutions. It may also facilitate standardization of procurement specifications and processes, particularly for high-cost and specialized medical products such as oncology drugs, dialysis supplies, and surgical equipment [18]. Furthermore, the use of national formularies and standardized tendering procedures is expected to enhance transparency and promote the rational selection of health commodities in accordance with the principles of the World Health Organization (WHO) on good governance in pharmaceutical procurement [19,20]. These principles are also reflected in Saudi Arabia’s National Medicines Policy, which aims to ensure the availability, affordability, quality, and rational use of medicines [19,20,21].

To support the centralized model, Saudi Arabia has adopted standardized procurement documentation, digital tendering platforms, and prequalification systems for suppliers. These initiatives provide a framework for strengthening oversight, improving transparency in procurement processes, and facilitating coordination across healthcare institutions. These reforms were formalized through agreements under which the Ministry of Health allocated its procurement budgets to NUPCO and transferred ownership of its main medical stores, and NUPCO partnered with the Expenditure Efficiency Center to improve the efficiency of government health spending [22].

However, the potential efficiency gains from centralized procurement must be considered alongside the risks associated with reduced purchasing flexibility and supplier concentration. In a survey of 79 pharmaceutical supply chain professionals in Saudi Arabia, 69.6% perceived centralized procurement as having a negative effect on the supply of essential medicines [12]. Reported concerns included the procurement of unregistered products, suppliers’ failure to deliver requested quantities, and inadequate consideration of previous product recalls. Although these findings reflect professional perceptions and cannot establish that centralized procurement caused medicine shortages, they identify potential weaknesses requiring further evaluation. Centralization may improve purchasing efficiency under stable supply conditions but become less responsive when urgent needs arise outside established tender cycles or when contracted suppliers cannot meet demand [23]. Maintaining prequalified alternative suppliers, allowing emergency purchasing at the facility level, and monitoring supplier performance could help balance purchasing efficiency with supply continuity. However, the effectiveness and costs of these measures in Saudi Arabia have not been independently evaluated.

### 4.2. Digital Transformation

The adoption of digital tools has the potential to improve procurement transparency, strengthen inventory oversight, and support data-driven planning across the healthcare system [6]. These reforms align with global efforts to digitize health logistics and strengthen supply network resilience, particularly in response to evolving global health challenges [24].

A key milestone is the deployment of NUPCO’s comprehensive e-procurement platform. This system digitizes the entire procurement cycle, from supplier registration and tender announcements to bid evaluation, contract management, and payment processes. By automating manual tasks and enabling digital monitoring, the platform is intended to streamline procurement processes, strengthen supplier accountability, and simplify reporting [25].

Beyond procurement, the Health Electronic Surveillance Network (HESN) and other digital health systems have supported public health surveillance, immunization monitoring, and emergency coordination [26]. However, these activities do not establish improvements in procurement, inventory management, or supply chain resilience. During the COVID-19 pandemic, national digital platforms, including HESN and the Tawakkalna application, supported case monitoring and the verification of individuals’ infection and vaccination status, and health information technologies were widely used for consultation, screening, and monitoring [27,28]. At the product level, the SFDA’s Drug Track and Trace System (RSD) traces medicines from manufacture to consumption, supporting supply chain monitoring and the real-time interception of recalled products, while predictive analytics and artificial intelligence are increasingly explored to support demand forecasting and reduce waste [29,30].

### 4.3. Emergency Preparedness and Pandemic Response

In Saudi Arabia, the COVID-19 pandemic revealed both vulnerabilities and strengths across various sectors, prompting a rapid, coordinated, and largely effective national response [31]. At the onset of the pandemic, Saudi Arabia mobilized its supply chain systems to procure and distribute essential items such as PPE, ventilators, diagnostic kits, and pharmaceuticals. Emergency procurement processes were accelerated to secure high-demand commodities from local and international markets. Nevertheless, the availability and prices of some essential medicines and PPE were affected during the early phase of the pandemic [32]. One of the key logistics developments was the national COVID-19 vaccination program, which required rapid expansion of vaccine storage, distribution, and vaccination-site capacity across regions, supported by digital registration and appointment platforms [33].

Empirical evidence from Saudi medical laboratories illustrates the limits of existing supply chain preparedness. A cross-sectional study involving 12 hospitals and 10 suppliers during the COVID-19 pandemic reported disruptions in procurement and inventory management, with lead times affected in 58% of hospitals, purchasing in 33%, and inventory management in 25% [34]. Reagent unavailability was the most frequently reported disruption (50%), highlighting the vulnerability of essential diagnostic services to interruptions in specialized supply chains. Although participating organizations reported using measures such as backup supply arrangements, recovery plans, and emergency agreements, these measures did not eliminate reported disruptions. The findings suggest that emergency preparedness requires not only coordinated procurement and distribution but also reliable access to alternative suppliers and critical materials. However, the small study was not designed to determine whether centralized procurement or dependence on imported inputs caused the reported shortages.

Saudi Arabia’s collaboration with WHO, framed through the WHO country cooperation strategy, has also supported the strengthening of emergency preparedness planning [35].

### 4.4. Local Manufacturing and Supply Chain Resilience

Per a commercial market forecast, Saudi Arabia’s cold-chain pharmaceutical logistics market was valued at approximately USD 143 million (SAR 537 million) in 2025 and is projected to reach about USD 318 million (SAR 1.19 billion) by 2034, a compound annual growth rate of 9.3% over 2026–2034. The e-pharmacy market, which reached about USD 1.09 billion (SAR 4.07 billion) in 2025, has further increased the need for reliable cold-chain logistics [36].

This growth is largely driven by increased public investment in healthcare infrastructure, the rising prevalence of chronic diseases such as diabetes, and the expanding role of e-pharmacies. With more than 23% of the adult Saudi population affected by diabetes [37], the demand for temperature-sensitive pharmaceutical products like insulin, vaccines, and biologics has increased substantially. In February 2025, Saudi Arabia inaugurated its first local insulin manufacturing facility, a joint venture between NUPCO, Sanofi, and Sudair Pharma Company. The facility is expected to produce approximately 15 million insulin pens per year, meeting 70% of the national demand and covering the needs of 500,000 patients with diabetes [38].

Additionally, according to news reports, NUPCO has secured SAR 2.5 billion in supplier financing through agreements with Tameed, First Abu Dhabi Bank, and Banque Saudi Fransi, alongside memoranda of understanding with logistics partners including Aramex, SMSA Express, DHL, and UPS to expand last-mile delivery. These arrangements aim to improve suppliers’ cash flow, including that of SMEs, and to expand distribution capacity [39].

Industry news and consultancy reports also describe a surge in demand for modern warehousing, particularly temperature-controlled and Grade-A facilities, with institutional investors and global logistics firms such as Moller–Maersk, DP World, and Arcapita funding cold-chain hubs and fulfillment centers in Riyadh, Jeddah, and Dammam [40]. One consultancy report valued the broader logistics and warehousing market at SAR 75 billion (USD 20 billion) in 2023, with projected annual growth of around 6.5% through 2030, and reported a 190% rise in transport and storage business licenses in Madinah from 2019 to 2024 [41].

While localization initiatives may reduce dependence on imported finished pharmaceutical products, they do not necessarily eliminate upstream supply chain vulnerabilities. A study of registered pharmaceutical products in Saudi Arabia found that 22.55% were manufactured domestically, illustrating the continuing importance of international production networks [9]. However, the location of finished-product manufacturing does not establish the origin of active pharmaceutical ingredients (APIs), excipients, packaging materials, or specialized manufacturing technologies. Domestic manufacturers may therefore remain vulnerable to international supply interruptions even when final production takes place within Saudi Arabia [10,42]. Current localization initiatives, including the establishment of insulin manufacturing capacity, represent important industrial developments, but their contribution to supply chain resilience cannot be determined from production targets alone. A more comprehensive localization strategy would require identifying critical imported inputs, diversifying upstream suppliers, and assessing the feasibility of domestic API production for selected essential medicines. These measures should be evaluated against their costs and expected contribution to supply security rather than assuming that greater domestic production automatically ensures pharmaceutical independence.

## 5. Interactions Between Components and Implications for Resilience

In our view, the contribution of these reforms to supply chain resilience depends less on each reform individually than on how they interact. Centralized procurement may provide a platform for coordinating other reform initiatives. By aggregating public-sector demand, NUPCO may provide more predictable purchasing volumes that could encourage domestic manufacturing, although effects on supply security have not been established. This potential policy linkage is illustrated by NUPCO’s partnership in the local insulin facility [38], its financing for local suppliers [39], and LCGPA’s use of government procurement to prioritize locally manufactured products [14]. Standardized specifications and unified tendering have also made national e-procurement data possible [25]. National digital platforms also supported COVID-19 surveillance and vaccination-related activities [27,28,33], but the available evidence does not demonstrate that these systems improved procurement, inventory management, or supply chain resilience. However, weaknesses in supply chain management and the absence of early notification of shortages have long contributed to medicine shortages [11], and in our view this connecting role is only partially realized, particularly between the public and private sectors.

The interaction between centralized procurement and emergency preparedness illustrates an important trade-off between efficiency and resilience. Consolidating demand through a national purchasing system may improve negotiating power, standardization, and coordination, but concentrating purchasing decisions or relying on a limited number of suppliers can also create common points of failure during disruptions [23]. Evidence from Saudi Arabia raises concerns about this balance. Supply chain professionals have reported procurement-related factors contributing to essential medicine shortages [12], while laboratory reagent shortages during COVID-19 demonstrated continuing vulnerabilities despite the presence of organizational resilience measures [34]. Although these studies do not establish a causal relationship between centralization and shortages, they indicate that purchasing efficiency alone is insufficient to ensure supply continuity. The appropriate degree of centralization may therefore differ by product criticality, availability of alternative suppliers, and urgency of clinical demand. Essential medicines and diagnostic supplies with limited substitutes may require dual sourcing, strategic reserves, or defined emergency purchasing exemptions, even when these arrangements increase routine procurement costs. Evaluating such arrangements requires considering both financial efficiency and the consequences of supply interruptions.

Similar trade-offs apply to pharmaceutical localization. Domestic manufacturing can reduce dependence on imported finished products and may improve responsiveness to changes in national demand. However, these benefits may be limited when APIs, raw materials, specialized equipment, or manufacturing technologies remain dependent on international suppliers [10,42]. Under these conditions, disruptions may shift from finished-product imports to upstream inputs without substantially reducing overall supply risk. Localization policies should therefore distinguish between final-product manufacturing and the domestic availability of critical production inputs. For strategically important medicines, combining selective upstream localization with diversified international sourcing and contingency inventories may provide greater protection than expanding domestic production alone. However, comparative evidence on the costs and resilience benefits of these approaches remains limited in Saudi Arabia. The proposed relationships should consequently be regarded as policy considerations requiring empirical evaluation rather than demonstrated effects of current reforms.

**Figure 1:**
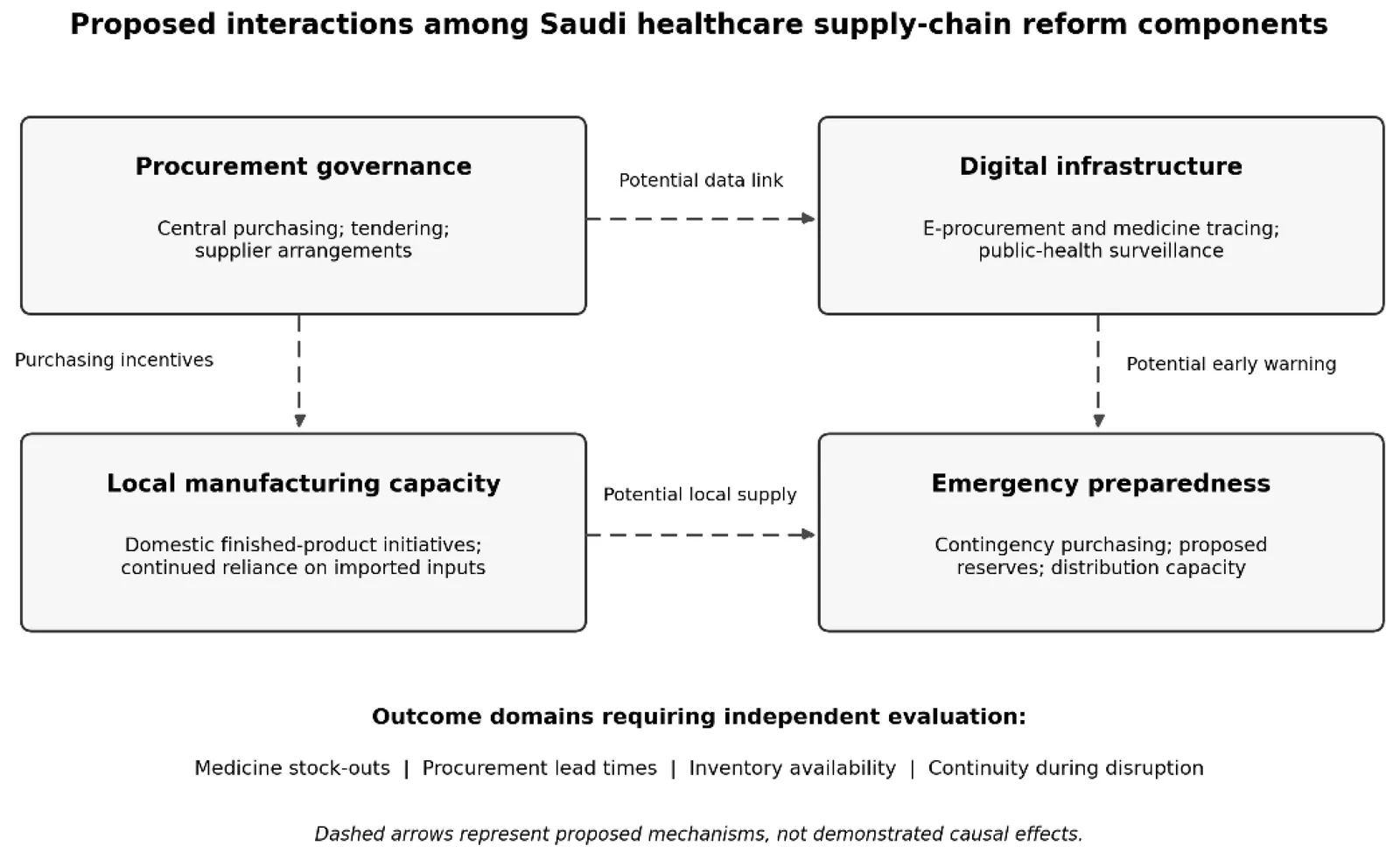
Conceptual framework of proposed interactions among healthcare supply chain reform components in Saudi Arabia.

Figure 1 summarizes the proposed interactions between the four reform components and identifies supply chain performance outcomes that require further empirical evaluation.

## 6. Applying the Framework: Antimicrobial Resistance

To illustrate how these interactions operate in a specific policy area, we apply the framework to AMR, which is used here as an application case rather than as a separate component, showing how procurement governance, digital infrastructure, local manufacturing, and emergency preparedness jointly shape the availability, quality, and appropriate use of antimicrobials.

The healthcare supply chain plays a pivotal role in both the containment and propagation of AMR, particularly through its influence on antimicrobial quality, availability, and use. In Saudi Arabia, recurrent shortages of essential medicines, linked to supply chain management weaknesses, procurement policies, and heavy reliance on imports [11,12], may reduce the availability of appropriate antimicrobials and push prescribers toward less suitable alternatives, undermining antimicrobial stewardship.

NUPCO oversees centralized procurement of antimicrobials across public institutions, and the SFDA’s track-and-trace system provides product-level traceability that can help detect counterfeit or recalled antimicrobials and monitor their movement through the supply chain [30].

Supply chain governance is also part of the national response to AMR. Under the first National Action Plan (2017), an initiative to strengthen the pharmaceutical supply chain, including procurement, supply, and management systems, led SFDA to establish a quality management system for the supply of medicines covering storage, transport, and expiry. In the second plan (2022–2025), led by the Public Health Authority, SFDA and NUPCO are supporting sectors for reporting on antimicrobial use [43]. In parallel, NUPCO’s electronic tendering and supplier platforms provide a channel through which local manufacturers, including producers of antimicrobials, can participate in public procurement, which could strengthen drug security and reduce the risk of antimicrobial shortages during global crises [25].

LCGPA plays an increasingly important role in strengthening health supply chains in Saudi Arabia, with implications for AMR prevention and control. Established in 2018, the authority is mandated to enhance national self-sufficiency by prioritizing locally manufactured goods, services, and technologies within government procurement systems. As its scope expands to include pharmaceuticals, medical devices, and essential health commodities, LCGPA is intended to reduce reliance on imported antimicrobials and critical medical supplies [14].

HESN, the national electronic surveillance network, provides a platform that could be extended to link antimicrobial stewardship data with information on prescribing, resistance trends, and supply bottlenecks [26]. Such integration would align with the One Health framework by linking pharmaceutical logistics with infection control and veterinary medicine surveillance. These measures are policy interventions whose effects on antimicrobial use have not been evaluated. To our knowledge, no study in Saudi Arabia has assessed whether centralized procurement, track-and-trace systems, or localization have reduced inappropriate antimicrobial use, antimicrobial shortages, or resistance. The links described in this section should therefore be read as plausible mechanisms rather than demonstrated effects, and evaluating them is a priority for future research.

## 7. Challenges

Several important challenges continue to affect the performance and resilience of Saudi Arabia’s healthcare supply chain system. The first is limited integration of supply chain data across different health sectors, particularly between public and private providers. There has also been no mandatory early notification of anticipated shortages, limiting the information available for coordinated responses [11]. This fragmentation may reduce system-wide visibility and delay coordinated procurement and distribution decisions during emergencies. It also constrains forecasting, because effective forecasting models require high-quality, standardized, and interoperable data across healthcare sectors. In terms of the framework, it weakens the link through which digital infrastructure connects procurement with distribution and emergency response. In the short term, SFDA, in coordination with NUPCO and the Ministry of Health, could introduce standardized early shortage reporting for public and private healthcare providers, including information on product availability and anticipated supply disruptions. Sharing these reports through existing systems could improve coordination across sectors without requiring additional digital infrastructure.

Supply chain professionals identify the procurement of unregistered medications, suppliers’ failure to deliver requested quantities, manufacturing problems, and poor demand forecasting as leading causes of shortages [12]. Continued reliance on a small number of international suppliers, especially for specialized pharmaceuticals, vaccines, and medical devices, further exposes the health system to global disruptions such as geopolitical tensions, trade restrictions, or pandemics. This weakens the link between emergency preparedness and upstream supply, because stockpiles and distribution capacity cannot fully compensate for disruption at the source.

Another emerging challenge is the presence of unregistered medical products and products without local regulatory representation or authorized distributors. These items often fall outside standard procurement and oversight pathways, posing risks related to quality assurance, traceability, and post-market surveillance. Their lack of formal regulatory status may also hinder timely supply replenishment, complicate forecasting accuracy, and introduce vulnerabilities into emergency procurement operations. Weak penalties for violations by manufacturers, importers, and distributors further limit enforcement [11]. These gaps may affect product traceability and reduce confidence in supply chain oversight mechanisms, weakening the oversight function of procurement governance itself.

In our experience, workforce capacity gaps in supply chain management may further limit efficiency. Despite Vision 2030′s emphasis on digital health and logistics modernization, there is still a shortage of professionals trained in procurement, data analytics, cold chain monitoring, and regulatory compliance, especially outside major urban centers. Limited technical capacity can slow the implementation of digital logistics systems and reduce the effectiveness of procurement reforms. Because these gaps affect procurement, digital systems, and manufacturing at the same time, they act as a cross-cutting constraint on all four components.

Regulatory harmonization is another area requiring attention. In our experience, differences in registration procedures and timelines for local versus imported products can create bottlenecks for market access. Streamlining approval processes and aligning with international standards could help accelerate local manufacturing scale-up. Delays in regulatory alignment may also discourage private sector investment in domestic pharmaceutical production, and they weaken the link between procurement governance and local manufacturing, because procurement preferences for local products cannot take effect until those products are approved.

In our experience, the main gap in performance monitoring concerns national standardization and public access rather than measurement alone. Some indicators already exist: NUPCO monitors the availability of critical items and order fulfilment for the public sector it supplies; the SFDA’s track-and-trace system generates product-level movement data [30]; and national reporting of antimicrobial consumption is included in the AMR action plan [43]. However, these indicators are not published in a form that allows comparison over time or across regions. Indicators that are largely missing at the national level include standardized measures of stock-outs, delivery lead times, inventory turnover, wastage, and cold-chain breaches, particularly indicators that cover both public and private providers. Without such indicators, it remains difficult to evaluate procurement efficiency, distribution equity, and system responsiveness across regions. Agreeing on common national definitions and publishing a core set of indicators would therefore be more important than creating new data systems.

Taken together, these challenges suggest that current reforms have not yet achieved full system-wide integration or performance optimization. In particular, the absence of unified national performance indicators limits the ability to assess whether recent investments have translated into measurable improvements in medicine availability, procurement timelines, or distribution efficiency. Addressing these evaluation gaps may help ensure that logistics modernization produces sustained health system benefits.

## 8. Lessons from the Saudi Case

Beyond the national context, the Saudi experience offers several insights for other countries pursuing state-led health supply chain reform, particularly high-income and centralized health systems in the Gulf region. First, centralized procurement can do more than reduce costs. When combined with industrial and digital policy, public purchasing power can be used to shape domestic markets and build national data systems. The value of centralization therefore depends on whether it is used as a policy instrument rather than only as a purchasing mechanism. Second, efficiency and resilience are not the same objective. Consolidating demand and suppliers may lower costs in normal times but concentrate risk during disruptions [23]. Resilient systems require deliberate redundancy, such as diversified suppliers, strategic reserves, and retained facility-level flexibility. This redundancy should be planned and financed as part of reform rather than treated as inefficiency. Third, financial resources alone do not guarantee supply independence. Even in a well-resourced system, localizing finished products leaves dependence on imported APIs, technology, and specialized expertise [42]. Localization strategies should therefore be selective and prioritize products of the greatest strategic importance. Finally, reforms cannot be shown to improve resilience without systematic measurement. Because the indicators that exist are rarely standardized or published, much of the progress described in this review is documented through policy announcements and institutional reports rather than independent evaluation. Building transparent performance monitoring into reforms from the outset would allow countries to identify which interventions work and to adjust them over time.

Of note, the contribution of the Saudi case is not to introduce new concepts, but to illustrate how centralized procurement, industrial localization policies, digital systems, and emergency preparedness initiatives are being developed within the same national reform program. It also highlights the difficulty of assessing the combined effects of these initiatives when standardized public performance indicators and cross-sector data integration remain limited. The relevance of these lessons to other countries will depend on differences in procurement structures, healthcare financing, regulatory capacity, and domestic manufacturing capabilities.

## 9. Limitations

This review has several methodological and evidence-related limitations that affect the ability to evaluate the performance impact of recent supply chain reforms quantitatively. First, it is based on a narrative review design and depends mainly on published articles and official policy documents. Detailed operational performance indicators, such as procurement cycle times, supplier performance metrics, logistics costs, inventory turnover rates, stock-out frequencies, delivery lead times, and cost-effectiveness measures, are not routinely available in the public domain. The limited availability of these data restricted our ability to conduct a quantitative assessment of supply chain performance and limited comparisons across institutions and time periods. Second, several major reforms under Vision 2030 remain ongoing, and their long-term impact cannot yet be fully evaluated. Third, because sources were selected purposively and several reforms are documented mainly in institutional reports, news articles, and commercial market forecasts rather than independent evaluations, some achievements may be overstated, and the interpretation reflects the perspective of authors working within the institutions discussed. Fourth, commercial market reports and news sources carry specific risks of bias. Market reports are produced for commercial purposes, rarely disclose their methods or data sources, may overstate growth, and are frequently revised; the market figures cited in this review, for example, changed in base year and forecast period between versions of the same report. News reports of agreements and facility openings describe announced intentions rather than implemented or evaluated outcomes and may reflect the perspective of the organizations involved. For these reasons, such sources were used only to provide context and were not used to support conclusions about supply chain performance or resilience. Fifth, the available studies provide information on perceived procurement weaknesses, medicine shortages, and disruptions in laboratory supply chains, but they do not establish the independent effects of centralized procurement on supply continuity. Similarly, publicly available evidence is insufficient to determine the extent to which domestic pharmaceutical manufacturing relies on imported APIs and other critical inputs, or whether localization initiatives have reduced these dependencies. Consequently, the relative benefits of centralized procurement, supplier diversification, strategic reserves, and upstream localization cannot be quantified from the available evidence. These limitations restrict the conclusions that can be drawn about the resilience effects of current reforms. Sixth, the proposed interactions between reform components have not been empirically evaluated and should not be interpreted as evidence of causal improvements in supply chain resilience.

## 10. Implications for Future Research

Several priorities emerge for future research. First, the effect of centralized procurement on medicine availability should be evaluated using national stock-out and procurement data, for example, through interrupted time-series analyses before and after the expansion of NUPCO. Second, the costs and supply-security benefits of localization should be compared with those of diversifying import sources, particularly for products that depend on imported APIs. Such evaluations should examine supplier concentration, the origin of critical manufacturing inputs, the availability of alternative suppliers, and the costs of maintaining strategic reserves or emergency procurement arrangements. Third, studies should assess interoperability between public and private supply chain data systems and its effect on visibility during emergencies. Fourth, simulation or stress-testing models could examine how the system would respond to future disruptions, such as a pandemic or the failure of a major supplier. Fifth, a national assessment of the supply chain workforce would help quantify skills gaps, particularly outside major cities. Finally, linking procurement and track-and-trace data with antimicrobial consumption data, for example using the WHO AWaRe classification, could show how supply chain governance affects antimicrobial use. Comparative studies with other Gulf countries implementing similar reforms would help identify which features of the Saudi model are transferable.

## 11. Conclusions

Saudi Arabia has introduced major healthcare supply chain reform initiatives involving centralized procurement, digital surveillance and traceability, cold-chain expansion, and local pharmaceutical manufacturing. Our analysis suggests that interactions among these initiatives may influence resilience, although limited public–private data integration, continued dependence on imported raw materials, regulatory bottlenecks, and workforce gaps could constrain their potential benefits. Sustaining progress will require deliberate investment in redundancy, selective localization, stronger data integration, and transparent performance indicators that allow reforms to be evaluated independently. However, the proposed relationships between these reforms remain conceptual and should not be interpreted as demonstrated causal effects.

## Figures and Tables

**Table 1:** Core components of health supply chain transformation in Saudi Arabia under Vision 2030: reforms implemented, expected benefits, and outcomes documented to date.

Element	Reforms Implemented	Expected Benefits	Documented Outcomes (Type of Evidence)	Remaining Limitations	Policy Implications
Procurement governance	Establishment of NUPCO; centralized procurement; transfer of MoH procurement budgets and main medical stores to NUPCO; standardized tenders and supplier prequalification	Greater purchasing efficiency and negotiating power; standardized specifications; more transparent procurement	No published independent evaluation of effects on availability or cost identified. Most surveyed supply chain professionals perceived centralized procurement as contributing to medicine shortages (survey)	Reduced institutional flexibility; concentration of risk in a single procurement structure; limited integration with private-sector procurement	Define a facility-level emergency purchasing pathway for items outside NUPCO tender cycles; extend NUPCO’s unified catalogue and procurement data standards to private providers
Digital infrastructure	NUPCO e-procurement platform; HESN; national COVID-19 digital platforms; SFDA RSD track-and-trace system	Transparency; product-level traceability; data-driven planning and demand forecasting	Implementation of NUPCO’s e-procurement platform and SFDA’s RSD system is documented through institutional sources. However, independent evaluations of their effects on procurement efficiency, inventory availability, medicine shortages, or supply chain resilience were not identified	Limited interoperability across sectors; incomplete national data integration	Link NUPCO procurement and inventory data with SFDA track-and-trace (RSD) movement data in a national supply dashboard covering public and private providers; make early shortage notification to SFDA mandatory
Emergency preparedness	Accelerated emergency procurement during COVID-19; national vaccination program with expanded storage, distribution, and vaccination-site capacity	Rapid mobilization of supplies and vaccines; capacity to absorb future shocks	Availability and prices of some essential medicines and PPE affected early in the pandemic (cross-sectional study); lead times affected in 58% of laboratories, and 75% adopted resilience measures (cross-sectional study)	Continued reliance on international suppliers; limited upstream surge manufacturing capacity	Agree on a national list of critical medicines and supplies for strategic reserves, reviewed jointly by MoH, the Public Health Authority, and NUPCO; build surge-capacity clauses into NUPCO contracts with local producers
Local manufacturing capacity	First local insulin manufacturing facility (2025); LCGPA localization agreements; SAR 2.5 billion supplier financing	Insulin facility projected to meet about 70% of national insulin demand (projection); reduced import dependence	Not yet evaluated; continued dependence on imported APIs and technology transfer (review)	Dependence on imported raw materials and technology transfer; regulatory bottlenecks affecting scale-up	Align LCGPA’s mandatory local-product list with NUPCO multi-year purchase commitments for priority medicines (e.g., insulin, essential antibiotics); provide SFDA priority review for localized priority products; support API production for selected strategic medicines

APIs, active pharmaceutical ingredients; HESN, Health Electronic Surveillance Network; IT, information technology; LCGPA, Local Content and Government Procurement Authority; MoH, Ministry of Health; NUPCO, National Unified Procurement Company; PPE, personal protective equipment; RSD, Drug Track and Trace System; SAR, Saudi riyal; SFDA, Saudi Food and Drug Authority.

## Data Availability

No new data were created or analyzed in this study. Data sharing is not applicable to this article.

## Appendix A. Search Strings

PubMed (“Saudi Arabia”[MeSH Terms] OR Saudi*[tiab] OR KSA[tiab]) AND (“supply chain*”[tiab] OR “health logistics”[tiab] OR logistic*[tiab] OR procurement[tiab] OR “cold chain”[tiab] OR “Pharmaceutical Preparations/supply and distribution”[MeSH] OR “drug shortage*”[tiab] OR “medicine shortage*”[tiab] OR “Drug Industry”[MeSH] OR “pharmaceutical manufactur*”[tiab] OR “local production”[tiab] OR localization[tiab] OR ((“Drug Resistance, Microbial”[MeSH] OR “antimicrobial resistance”[tiab]) AND (procurement[tiab] OR “supply chain*”[tiab])))
Scopus TITLE-ABS-KEY (“Saudi Arabia” OR saudi OR ksa) AND TITLE-ABS-KEY (“supply chain*” OR “health logistics” OR procurement OR “cold chain” OR “drug shortage*” OR “medicine shortage*” OR “pharmaceutical manufactur*” OR “local production” OR localization OR (“antimicrobial resistance” AND (procurement OR “supply chain*”))) AND TITLE-ABS-KEY (health* OR medic* OR pharma* OR drug* OR vaccine*)
